# Impacts of El Niño-Southern Oscillation on the wheat market: A global dynamic analysis

**DOI:** 10.1371/journal.pone.0179086

**Published:** 2017-06-08

**Authors:** Luciano Gutierrez

**Affiliations:** Affiliation Department of Agricultural Sciences and Desertification Research Centre, University of Sassari, Sassari, Italy; Universidad Nacional de Mar del Plata, ARGENTINA

## Abstract

Although the widespread influence of the El Niño-Southern Oscillation (ENSO) occurrences on crop yields of the main agricultural commodities is well known, the global socio-economic consequences of ENSO still remain uncertain. Given the global importance of wheat for global consumption by providing 20% of global calories and nourishment, the monitoring and prediction of ENSO-induced variations in the worldwide wheat market are essential for allowing national governments to manage the associated risks and to ensure the supplies of wheat for consumers, including the underprivileged. To this end, we propose a global dynamic model for the analysis of ENSO impacts on wheat yield anomalies, export prices, exports and stock-to-use ratios. Our framework focuses on seven countries/regions: the six main wheat-exporting countries—the United States, Argentina, Australia, Canada, the EU, and the group of the main Black Sea export countries, i.e. Russia, Ukraine, and Kazakhstan—plus the rest of the world. The study shows that La Niña exerts, on average, a stronger and negative impact on wheat yield anomalies, exports and stock-to-use ratios than El Niño. In contrast, wheat export prices are positively related to La Niña occurrences evidencing, once again, its steady impact in both the short and long run. Our findings emphasize the importance of the two ENSO extreme phases for the worldwide wheat market.

## Introduction

In November 2016 the National Oceanic and Atmospheric Administration (NOAA) issued a warning that weak La Niña conditions were observed during October 2016. These conditions are likely to persist during the winter of 2016–2017. La Niña and El Niño conditions are opposite phases of what is known as the El Niño-Southern Oscillation (ENSO) cycle. ENSO is a cyclical ocean-atmosphere phenomenon which originates in the tropical Pacific. The El Niño phase is a periodic warming of sea surface temperatures across the central and central east equatorial Pacific. While El Niño brings heavy rains to south-eastern South America, western North America and eastern Africa, it creates droughts in Australia, India and Indonesia. The opposite phase, known as La Niña, is a periodic cooling of ocean surface temperatures in the central and central east equatorial Pacific. La Niña spreads heavy rains in the western Pacific, including Australia, and a colder than average temperatures in Canada and the western and northern United States, [1, 2]. It is now clear that ENSO can trigger noticeable anomalies in rainfall and temperature patterns around the globe, including Europe [3, 4].

ENSO influences, through “teleconnections”, the global dynamics of seasonal winds, rainfall and temperature. Although the development and evolution of ENSO events and their impacts on crop yields of the main agricultural commodities are known [5, 6, 7, among others], the global socio-economic consequences of ENSO are still uncertain. [8] used several vector autoregressive (VAR) models to examine the historical effects of the ENSO cycle on world primary commodity prices and on some indicators of economic activity. He found that a positive shock to the ENSO variable raises real commodity price inflation between 3.5 and 4 percentage points. In addition, he found that ENSO appears to account for almost 20% of commodity price inflation movements. [8]’s evidence confirmed that ENSO influences world consumer price inflation and world economic activities. [9] used correlation and Granger causality tests to study the effects of the ENSO phenomenon upon 22 economies and their business cycles. They found that the ENSO impact is much weaker compared to the study by [8]. Their main result shows that El Niño has relatively few detectable effects on the business cycles of most of the countries, with the exceptions of South Africa, Australia and India. More recently, [10] employed a dynamic multi-country model to analyze the international macroeconomic transmission of ENSO weather shocks. Their results highlight that there are considerable heterogeneities in the responses of different countries to ENSO-positive (i.e. El Niño) shocks. Notably, while some countries experience short-lived falls in economic activity in response to the event, for other countries (the United States and the European region), the El Niño occurrence has a growth-enhancing effect. In addition, most countries experience short-run inflationary pressures as both energy and non-fuel commodity prices increase. Other researchers have examined the microeconomic impact of ENSO on commodity prices such as vegetable oil and coffee, [11–12]. [11] demonstrated the asymmetric effects of El Niño and La Niña events on coffee prices and [12] found that El Niño events result in the increase of vegetable oil prices.

Given the importance of wheat in providing more nourishment for human consumption than any other food source, the monitoring and prediction of ENSO-induced variations on wheat yields, export prices and stocks are essential in ensuring that national governments have sufficient supplies of wheat for consumers, including poor segments of the population. Wheat is the leading source of vegetable protein providing around 20% of world calories for human consumption. In 2014, more than 221 million hectares were planted and produced approximately 174 million metric tons. The global wheat trade is close to that of maize and rice combined (184 million tons in the marketing year 2015–2016). In order to fill the knowledge gap of ENSO variations and effects on the worldwide wheat market, we propose a global dynamic model to investigate the impact of ENSO occurrences on wheat yields, export prices, stocks and exports.

Since interdependencies exist among different countries and regions, a global dynamic model allows us to take into account both the temporal and cross-sectional dimensions of wheat data. We think this is an important feature as we expect that ENSO occurrences affect both the domestic and the global wheat markets. Specifically, we use a new version of the global vector autoregressive (GVAR) model presented by [13] to analyze the impacts of El Niño and La Niña on wheat export prices, yield anomalies, stocks and exports. Our framework focuses on seven country and region-specific models: the six main wheat-exporting countries and regions the United States, Argentina, Australia, Canada, the EU, the main Black Sea wheat-exporting countries of Russia, Ukraine and Kazakhstan (RUK) plus the rest of the world (ROW). We estimate a vector error correction model for each country/region, where the domestic endogenous variables of wheat yield anomalies, prices, stocks, exports and food prices are linked to the corresponding foreign variables to match the international relationships of the country or region under consideration. The individual models are then aggregated by using trade weights to generate the global dynamic model. To deal with the multiple aspects of ENSO, it has been suggested [14] that more than one index could be combined into a composite index. The Multivariate ENSO Index (MEI) links six different ENSO indicators and is considered as one of the most representative measure of ENSO events [15]. In each of the individual models, we include a measure of the intensity of ENSO occurrence through the MEI measure. Positive values of the MEI represent the warm ENSO phase, i.e. El Niño, while negative MEI values represent the cold ENSO phase, i.e. La Niña. Exogenous shocks connected to El Niño or La Niña anomalies can be easily taken into account in the GVAR model which admits for direct, or first-round, effects on economies of individual countries as well as second-round effects originating from the international market, thus allowing for complex interactions and interdependency at a variety of levels (domestic and international).

The results show that ENSO occurrence affects all export countries, and it has differentiated relevant first and second-round effects in the short as well as the long run. El Niño and La Niña exert asymmetric effects on wheat yields, prices, stock-to-use ratios and exports. At the global level, El Niño events have negative impacts on wheat yields (especially during the first semester), with Australia experiencing the highest negative impact. Among the main wheat exporters, however, Argentina and the EU are exceptions, showing positive reactions to the shocks. La Niña, on the other hand, creates an average a stronger negative effect than El Niño, with the exception of the US, which presents an increased wheat yield in the first semester. From observing wheat export prices, it is evident that La Niña presents positive, significant and steady effects, with exports and stocks mimicking the yield movements.

## Background

It is now recognized that atmospheric and oceanic cycles can induce climate anomalies with several studies emphasizing the role of two extreme anomalies, El Niño and La Niña, in influencing agricultural commodity production [16, 17, 18, 19, 20, 7].

**Fig 1:**
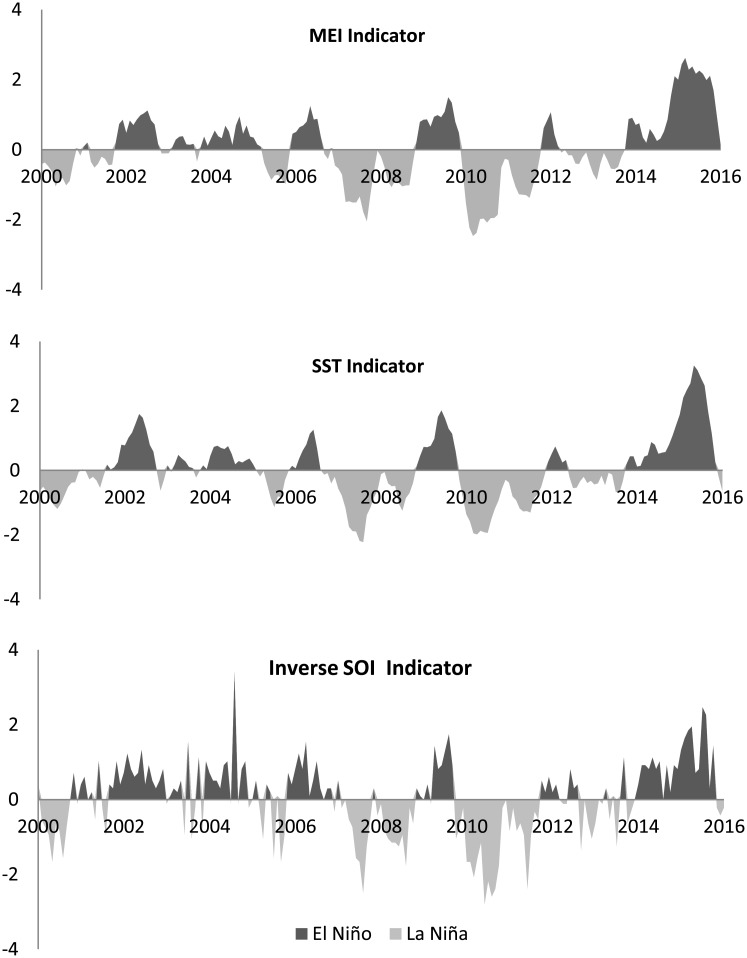
ENSO event indicators: El Niño years (black), La Niña years (grey). Standardized variables.

Different measures of ENSO are available. Some of them are presented in Fig 1. All the indicators are calculated as bimonthly averages. The first graph shows the Multivariate ENSO Index (MEI). It consists of the six main observed climatic variables in the tropical Pacific: sea-level pressure, zonal and meridional components of the surface wind, sea surface temperature, surface air temperature, and percentage of total cloud cover. The MEI is calculated as the first unrotated principal component of all six combined fields. This is accomplished by the initial normalization of the total variance of each field, followed by the extraction of the first principal component, [21]. While MEI takes into account the different indicators of climate anomalies, the other two ENSO measurements refer to single indicators. The Sea Surface Temperature (SST) anomalies are computed as deviations between sea surface temperatures in the 3.4 Pacific region (i.e. the central and central-eastern equatorial Pacific), and the region’s historical average. Following this indicator, El Niño and La Niña refer, respectively, to warmer or cooler sea temperatures in the 3.4 zone of the Pacific. The last index is the Southern Oscillation Index (SOI). It measures the differences in air pressure in the South Pacific (between Tahiti and Darwin). Deviations of the SOI from its historical averages indicate the presence of El Niño (the warm phase of the Southern Oscillation cycle) or La Niña (the cold phase of the Southern Oscillation cycle). A further measure is the Oceanic Niño Index (ONI) which uses the same data as SST but it is defined as a three-month running mean. In this scenario, to be classified as an El Niño or La Niña phase, the anomalies must exceed +0.5*C* or −0.5*C* for at least five consecutive months.

The previous indicators have been extensively used in the analysis of the impacts of ENSO anomalies mainly on crop yields and prices. In a recent paper, [7] used the ONI measure to provide global maps that describe ENSOs effects on some crop yields, including wheat. Analyses of yield anomalies (deviations from the five-year running mean) show significant negative impacts of El Niño on the wheat yields in parts of China, the United States of America, Australia, Mexico and parts of Europe. Positive impacts of El Niño are recorded in Argentina, Kazakhstan, and parts of South Africa. Significant negative impacts of La Niña appear in parts of North, Central and South America, and Ethiopia. The La Niña phase exerts significant positive impacts in parts of South and West Africa. In terms of the global mean, wheat yields in both El Niño and La Niña years tend to be below normal (-4.0 to -0.2%, [7]), with La Niña generally showing a stronger and negative impact. The main differences between [7] and our work are, firstly, that [7] presented grid-cell crop yields movements following ENSO anomalies at global level for the period 19822006. Due to lack of data for the main economic variables, we are only able to focus on the main wheat export countries and a Rest of World regions analyzed during a different period, 2000–2016. Moreover, we are more interested in the analysis of the dynamic responses of wheat yield anomalies, export prices, wheat exports, and stock-to-use ratio following an ENSO shock, rather than in providing a world map of the average impacts of ENSO on the wheat yields. Interestingly, despite the differences in the approach, the two studies show similar results with respect to the ENSO impacts on yields.

Given the severe effects of ENSO occurrence on crop yields, some studies have also analyzed its impact on commodity prices. Using home market models (i.e. without trade) and the SOI index, [22] shows that a statistically significant correlation exists between extreme ENSO occurrence and soya bean futures prices. However the same is not true for wheat and corn futures prices. Similar results have been shown by [9]. [12] examined the effects of El Niño on market dynamics of major vegetable oil prices. Using a smooth transition model they showed that El Niño events result in the vegetable oil price increasing, while La Niña occurrence results in a price decrease. [11] also demonstrated the asymmetric effects of El Niño and La Niña events on coffee prices.

In contrast to the previous works that focus on the analysis of country-specific markets, we employ a dynamic multi-country model to analyze effects of El Niño and La Niña occurrences on wheat markets, taking into account trade effects. We expect that a multi-country specification has many advantages. First, the model allow to examine the market fluctuations and interactions among countries. This is important given the world dimensions of wheat price dynamics that involve different countries. Secondly, it allows us to model for example the evolution of wheat export prices generated by possible shocks on domestic variables such as wheat yield, the nominal exchange rate, and input costs, and by the impacts of foreign variables—i.e, variables that are strictly linked to domestic variables that may influence the domestic economy. Finally, and most importantly, a dynamic multi-country model can take into account not only first-round impacts of ENSO events on the home markets but also second-round effects such as, for instance, export price propagation of shocks through the international markets.

## A GVAR-climate model

The dynamic multi-country model is mainly based on the Global Wheat Market Model (GLOWMM) proposed by [13]. Specifically, we use a global vector autoregression (GVAR) model that is particularly suited to the analysis of the channels of transmission of shocks from one country, or region, to others markets. As previously reported, we believe this is a fundamental characteristic that any commodity model must contain in order to allow analysis of how exogenous shocks, like an ENSO occurrence, impact different economies and spread around the world. The GLOWMM considers the six main export regions: the United States, Argentina, Australia, Canada, the EU, the group of Black Sea countries, Russia, Ukraine and Kazakhstan (RUK). A further region is given by the rest of the world (ROW) and is included in order to take into account for the effects exerted on the market by all other countries. For each country, we consider as endogenous variables the wheat export prices $p_{it}^{e}$ quoted in US dollars, the wheat stock-to-use ratio *z*_*it*_, defined as the sum of ending stocks divided by the total utilization, and the nominal exchange rate *e*_*it*_ defined as the local currency in region *i* per unit of US dollar. The index of consumer food prices is represented as $p_{it}^{c}$. This latter variable is used as a benchmark for food inflation in each region *i*. We also include the fertilizer price $p_{it}^{f}$ is defined in local currency. In this version of the paper, the total exports *xe*_*it*_ and the wheat yield anomalies *ya*_*it*_. This variable is obtained as deviations of the log of yield in each country or region from their trend cycles computed by the [23] filter. Supporting information section includes details and information on the dataset. The interested reader can refer to [24, 25], or [26] recent book on GVAR modelling, and [13] for a review of this research field of analysis.

The previous variables, excluding *ya*_*it*_ and *z*_*it*_, are the log of indexes with the base year July/2000-June/2001. Each country’s system of variables is also influenced by global exogenous variables such as the oil price $p_{t}^{o}$ and by the *ENSO*_*t*_ measurement. We focus the analysis on the Multivariate ENSO Index (MEI) measurements, which synthesize a larger number of meteorological variables that can affect the ENSO event. As we will see in the “Robustness Checks” section, although the signs and shapes of the effects of SST or SOI anomalies on wheat prices are similar to the MEI indicator, some differences in the intensity of the effects have been observed. Specifically, the MEI and SST generally report similar impacts, while the SOI measurement shows minor effects on endogenous variables. One of the features of GVAR specification is that it can include what are called “foreign variables”. In our case, they are given by the average competitor prices $p_{it}^{e*}=\sum_{j\neq i}w_{j}p_{jt}^{e}$, the average stock-to-use ratio $z_{it}^{*}=\sum_{j\neq i}w_{j}z_{jt}$, the effective exchange rate $e_{it}^{*}=\sum_{j\neq i}w_{j}e_{jt}$, the average of the food price indexes $p_{it}^{c*}=\sum_{j\neq i}w_{j}p_{jt}^{c}$, the average yield anomalies and $ya_{it}^{*}=\sum_{j\neq i}w_{j}ya_{jt}$, $xe_{it}^{*}=\sum_{j\neq i}w_{j}xe_{jt}$ that is the average exports. The weights *w*_*j*_ are given by the average export weights computed for the wheat marketing years 2014-2016. Each foreign variable is defined using the constraint that ∑_*j* ≠ *i*_ *w*_*j*_ = 1, and *w*_*ii*_ = 0. The choice of trade weights is based on the motivation that exogenous shocks, including ENSO shocks, could be passed on in all countries or regions through the trade channel.

The GVAR model specification requires two steps. In the first step a vector autoregressive model with exogenous *X* variables, labelled *VARX*(*p*_*i*_, *q*_*i*_), is specified, where *p*_*i*_ and *q*_*i*_ are the number of lags of the endogenous and exogenous variables, and the index *i* indicates the country or region *i*.

Hence the *VARX*(*p*_*i*_, *q*_*i*_) for each country is specified as,

$$\begin{array}{c} \phi_{i}\left(L,p_{i}\right)y_{it}=a_{i0}+\Lambda_{i}\left(L,q_{i}\right)y_{it}^{*}+\Psi_{i}\left(L,q_{i}\right)x_{t}+\varepsilon_{it}\;\;\;i=1,\ldots ,N;t=1\ldots T, \end{array}$$ (1)

where *a*_*i*0_ is a (*k*_*i*_ × 1) coefficient vector of the deterministic intercept, with *k*_*i*_ the number of endogenous variables in country *i*; *y*_*it*_ is a (*k*_*i*_ × 1) vector of country specific variables and related (*k*_*i*_ × *k*_*i*_) matrices of lagged coefficients $\phi_{i}\left(L,p_{i}\right)=I-\sum_{p=1}^{p_{i}}\phi_{i}L^{i}$, where *L* is the lag operator. The variable $y_{it}^{*}$ is a $\left(k_{i}^{*}\times 1\right)$ vector of trade-weighted foreign variables and the corresponding $\left(k_{i}\times k_{i}^{*}\right)$ matrix lag polynomial denoted by Λ_*i*_(*L*, *q*_*i*_). Ψ_*i*_(*L*, *q*_*i*_) is a matrix lag polynomial connected to the global exogenous variables *x*_*t*_. The distinction between foreign variables *y*_*it*_ and the global exogenous variable *x*_*t*_ is important for the analysis of the dynamic and statistical properties of the GVAR model. Finally, *ε*_*it*_ is a (*k*_*i*_ × 1) vector of zero mean, serially uncorrelated country-specific errors with a time-invariant variance matrix *Σ*_*ii*_ i.e. *ε*_*it*_ ∼ *iid*(0, Σ_*ii*_). For inference and estimation purposes, we assume the weak exogeneity of $y_{it}^{*}$ and rules out long-run feedbacks from *y*_*it*_ to $y_{it}^{*}$. Estimation of Eq (1) is performed using a vector error correction specification (VECMX) in order to take into account the integration properties of the series. The VECMX country models are estimated separately for each country or region *i*. As is well known a VARX form can always be rewritten as a VECMX form, and vice versa.

[13] showed (see pg. 1497-1498) that, by stacking all individual vector autoregressions in Eq (1) and linking them by using a matrix of weights, it is easy to obtain the following global vector autoregression

$$\begin{array}{c} y_{t}=b_{0}+F_{1}y_{t-1}+F_{2}y_{t-2}+\ldots +F_{p}y_{t-p}+\Theta_{0}x_{t}+\Theta_{1}x_{t-1}+\ldots +\Theta_{q}x_{t-q}+v_{t} \end{array}$$ (2)

where *p* and *q* are the maximum value of lags among the country-units *i*. Eq (2) can be used to compute the marginal impact of the *x*_*t*_ exogenous variable on the vector of endogenous *y*_*t*_. To do this, it is useful to rewrite Eq (2) in a simpler GVAR(1,0) form [27] pp. 402-403,

$$\begin{array}{c} Y_{t}=C+AY_{t-1}+BX_{t}+U_{t} \end{array}$$ (3)

From Eq (3), by successive substitution for lagged *Y*_*t*_’s and using the matrix *J* = [I_*K*_ 0 ⋯ 0] of dimension (*K* × (*Kp* + *K*^*X*^*q*)), where *K*^*X*^ is the number of global exogenous variables in the model, to select all the endogenous variables, we obtain

$$\begin{array}{c} Y_{t+h}=JC\sum_{j=0}^{h-1}A^{j}+JA^{h}Y_{t}+\sum_{j=0}^{h-1}JA^{j}Bx_{t+h-j}+\sum_{j=0}^{h-1}JA^{j}J^{{}^{\prime }}v_{t+h-j}, \end{array}$$ (4)

and the coefficient matrices of the exogenous variables in the final form representation will be given by

$$\begin{array}{c} D_{j}=JA^{j}B,\;\;j=0,1,\ldots , \end{array}$$ (5)

Thus, the matrices *D*_*j*_ reflect the marginal impact, in the period *j*, of changes in the exogenous variables on the endogenous variables of the system. In other words, after solving the Global VAR model, the possible differential effects of El Niño and La Niña shocks on the endogenous variables of different countries can be examined using the multipliers Eq (5).

As previously reported, the GLOWMM allows for six country models that refer to the main export countries or regions, plus a further model for the rest of the world (ROW). These models are estimated separately at monthly frequency during the period June 2000 to June 2016.

As we have seen before, we consider the following set of endogenous variables

$$\begin{array}{c} y_{it}\equiv {\left[p_{it}^{e},ya_{it},xe_{it},z_{it},e_{it},p_{it}^{c},p_{it}^{f}\right]}^{\prime },\;\;i=0,1,\ldots , \end{array}$$ (6)

The *ya*_*it*_ variable is obtained as deviations of the log of yields in each country or region and their trend cycles are computed by the [23]. We use a smoothing parameter of *λ* = 129600, as suggested by [28] for monthly series. We do not include the exchange rate variable *e*_*it*_ in the US model as well as the export price in the ROW model that we assume is exogenously determined in the international wheat market [13]. Rather, export prices will influence the ROW region from the foreign-specific variable. In addition we include the foreign-specific variables constructed as (geometric) averages of the country-specific variables using as weights the wheat-exporting country’s shares, i.e. the average competitor price $p_{it}^{e*}$, the average wheat exports $xe_{it}^{*}$ and yields $ya_{it}^{*}$, the effective exchange rate $e_{it}^{*}$—i.e. the average of a country’s bilateral exchange rate—the average stock-to-use ratio $z_{it}^{*}$, and the average food price $p_{it}^{c*}$. Finally, each country’s system of variables is also influenced by global (exogenous) variables given by the world oil price $p_{t}^{o}$, and by the *ENSO*_*t*_ indicators. We define the *ENSO*_*t*_ anomalies and include them in the model through the deviation of the MEI index from the average values during the period 2000–2016 and divided by the standard deviations. Positive values, greater than 1, identify *ElNiño*_*t*_ anomalies and negative values, below -1, identify *LaNiña*_*t*_ anomalies. Thus the GLOWMM model allows us to analyze the domestic and international effects of a shock to *ElNiño*_*t*_ or *LaNiña*_*t*_ on wheat market variables.

## Results

**Fig 2:**
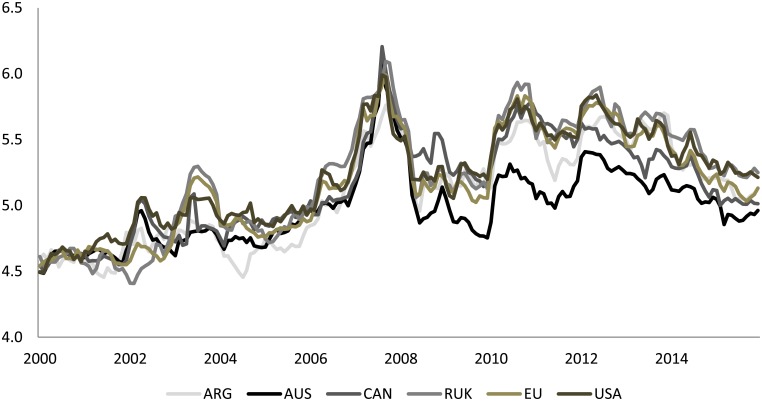
Logs wheat export prices: July 2000June 2016. Index July 2000–June 2001 = 100.

Before analyzing the results from the GVAR model, it is useful to introduce a brief analysis of the main data used in the paper. Moreover, we will focus on the correlations among El Niño, La Niña, extreme events and some economic endogenous variables which characterize the global wheat market, i.e. the average export prices, global yields, exports in volume and the average stock-to-use ratio. Table 1 provides a summary of the statistics. As shown in Fig 2, the wheat export prices in 2007-2008 were characterized by an astonishing rise (the wheat price reached its maximum in the main export countries during February-March 2008), followed by a sudden decline. Compared to the minimum value of the 2000-2016 period, the average wheat export prices in 2008 rose by 158%, see Table 1. The literature has proposed numerous factors to explain the commodity price movements, including growing food demand in developing countries, cost of inputs, weak US dollar, speculation in the commodity markets and adverse weather impacts [29]. In Table 1 we also included statistics on wheat yield anomalies (computed as log difference from yield trend-cycle), as well as exports and stock-to-use ratio. Interestingly, during the months between February and March 2008, severe yield anomalies, and export and stock-to-use ratio reductions were registered. Lastly, during the period of analysis, the variables reported in Table 1 were approximately symmetric given the low values of the second Pearson’s coefficients.

**Table 1:** Key economic indicators of the global wheat market.

Variables	Median	Std Dev.	Max	Min	Skewness^b^
Export prices^a^	5.16	0.38	6.06	4.48	0.03
Yields anomalies	0.90	8.39	17.04	-25.72	-0.27
Exports^a^	4.96	0.33	5.49	4.00	-0.07
Stock-to-use ratio	0.34	0.08	0.57	0.17	0.27

Notes:

^a^Logarithms of index, base 2000.7-2001.6 = 100.

^b^The skewness statistic is the second Pearson’s coefficient of skewness.

We then analyzed possible correlation among the previous variables and El Niño and La Niña extreme phases. To this end, we used percentiles. Specifically, values of the MEI indicator higher than 0.637 (the 70th percentile) may indicate El Niño moderate or strong events and values lower than −0.243 (the 30th percentile)denote strong or weak La Niña conditions. Values in the -0.243 to 0.637 interval were interpreted as ENSO neutral phase. Following the previous criteria, during the period of analysis between July 2000 and June 2016, 58 months were characterized by El Niño conditions and approximately the same number of months (57) were identified as La Niña extreme events. The remaining 78 months belong to the neutral phase. Then, we computed the deviations of the economic variables from their trend-cycles and selected these values according to the three ENSO’s phases. The correlation analysis among these variables and the three ENSO phases can give a first insight into the ENSO impacts on the wheat market. In Table 2 we noted that strong relationships with the economic variables were evidenced by La Niña extreme events. Intensification of La Niña events gave rise to a lower level of wheat yields and exports and increase in the wheat export prices. Although a negative correlation was reported in the Table 2, the stock-to-use ratio was not significantly influenced by La Niña conditions. El Niño events seemed to have minor effects at a global level. The correlation coefficients were generally not significant, with the exception of exports in volume. Similar results were reported for the ENSO neutral phase.

**Table 2:** Contemporaneous correlation between of El Niño, La Niña, and Neutral phases and main economic variables.

Variables	El Niño	La Niña	Neutral
Export prices	-0.12 (0.19)	0.39 (0.00)	-0.15 (0.26)
Yields	-0.13 (0.17)	-0.53 (0.00)	0.04 (0.33)
Exports	-0.44 (0.00)	-0.47 (0.00)	0.01 (0.49)
Stock-to-Use ratio	-0.03 (0.41)	-0.13 (0.17)	0.12 (0.28)
N. Observations	58	57	77

Notes: In parentheses the p-value statistics. La Niña variable has been multiplied by -1 to facilitate sign comparison.

Despite the previous results being interesting, as they evidenced possible relationships between ENSO events and economic variables, it is well known that the value of one economic variable is usually connected with its own lags and depends on the values of other variables. In other words, it is necessary to analyze the system of dynamic interrelationships between a number, sometimes many, of economic variables. The GVAR model can accomplish this task. This is crucial in the case of wheat market, given the global dimension, for example, of the commodity-price dynamics, and the interconnected economic movements in most of countries.

The first step of the GVAR model is the analysis of the non-stationary properties of the series in the model. We compute the ADF test for the null hypothesis of the non-stationarity of the series. The majority of the series are *I*(1), i.e. non-stationary, with the exception of the *ElNiño*_*t*_, *LaNiña*_*t*_, and yield anomaly measures for which we reject the null hypothesis of non-stationarity. The country-specific models are estimated subject to the reduced cointegration rank restrictions [30, 31].

For each country VARX model in Eq (1), we use the Akaike criterion to infer the *p*_*i*_ and *q*_*i*_ lag orders. We choose as maximum values *p*_*i*_ = 3 and *q*_*i*_ = 2. The Akaike criterion suggests values of *p*_*i*_ = 2, 3, see Table 3, while the *q*_*i*_ order is always equal to *q*_*i*_ = 1. Further, for each regression and in each country model, we compute tests for the residual autocorrelation using the modified LM statistic proposed in [32, 33]. On the whole, the results do not refuse the white-noise residual autocorrelations hypothesis.

**Table 3:** Order, case and number of cointegrating relationships.

Country	*p*_*i*_	*q*_*i*_	Case	Cointegrating Relationships
USA	3	2	(III)	3
ARG	3	1	(IV)	2
AUS	3	1	(III)	3
CAN	2	2	(III)	1
RUK	3	2	(III)	2
EU	2	2	(IV)	3
ROW	3	2	(III)	2

Notes: Rank orders are derived using Johansen’s trace statistics at the 95% critical value level. The VARX *p*_*i*_ and *q*_*i*_ orders are computed using Eq (1)

The previous results allow the estimation of the VARX eq (1) as a vector error correction specification (VECMX). We analyze how the deterministic component enters the model. We investigate two possible cases. In the first, we allow for an unrestricted intercept and no trend coefficients. We also allow for a model with a co-trending restriction or an unrestricted intercept [15]) and a test of whether the cointegrating relations are trended or not trended has been carried out. The deterministic setup used for each region—case III (unrestricted intercept and no trend coefficients) or case IV (unrestricted intercept and a co-trending restriction)—is presented in the fourth column of Table 3. The effects of shocks to the system model have been considered using persistence profiles. On impact, the persistence profiles are normalized and the rate at which they tend to zero provides information on the speed with which equilibrium correction takes place in response to shocks. The estimated persistence profiles initially overshoot, i.e they exceed the value of one, but they tend to zero highlighting the cointegration properties of the system. We note that the speed of convergence is very fast for all countries, with the US showing the lowest rate of convergence.

The cointegrating rank was computed using the maximal eigenvalue test [31], as set out in [34] for models with weakly exogenous *I*(1) regressors. The statistic is calculated at the 95% significance level for case III or case IV depending on the results obtained from a likelihood ratio test statistic. The case used for each region is also presented in Table 3. The number of cointegrating equation is reported in Table 3. In all cases, the exact identity matrix of normalized cointegrating vectors has been computed [27], pp. 249-250. For cointegrated models, the coefficients structural stability can be as relevant in the short run as it is the long run. We propose different structural stability tests to analyze for possible parameter instability, using [35] maximal OLS cumulative sum (CUSUM) statistic, and its mean square variant and [36] test. The results show parameter stability during the period of analysis.

**Fig 3:**
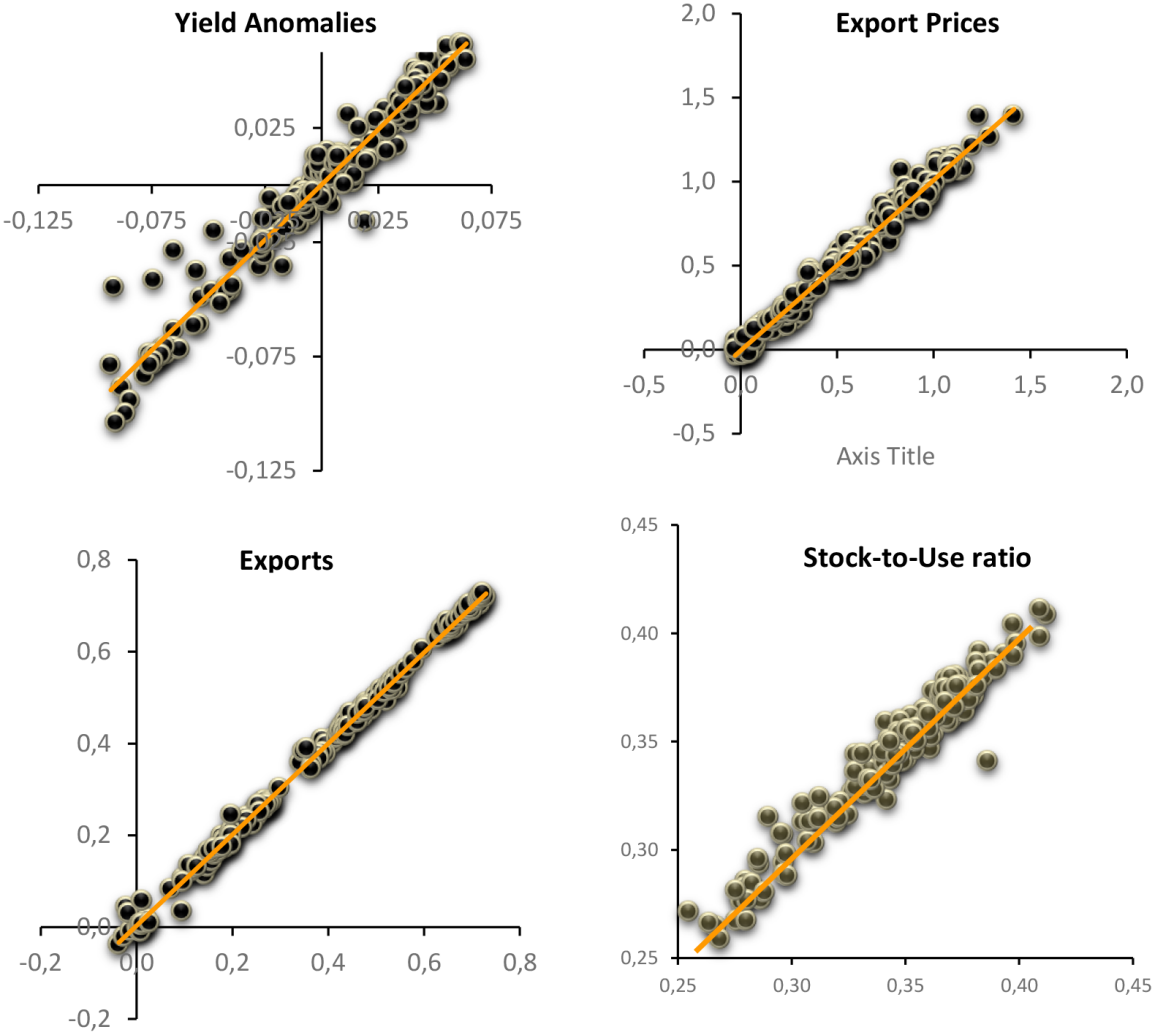
Scatter plot predicted (X-axis) and actual (Y-axis) values for some endogenous variables.

In Fig 3 we plot the predicted values (X-axis) versus the observed values (Y-axis) for yield anomalies, export prices, export and stock-to-use ratio. The GVAR model seems very accurate, showing a strong correlation between the models predictions and its actual results. The values of the correlation coefficient range from the lowest value of 0.972 for the stock-to-use variable and 0.998 for the export variable.

**Fig 4:**
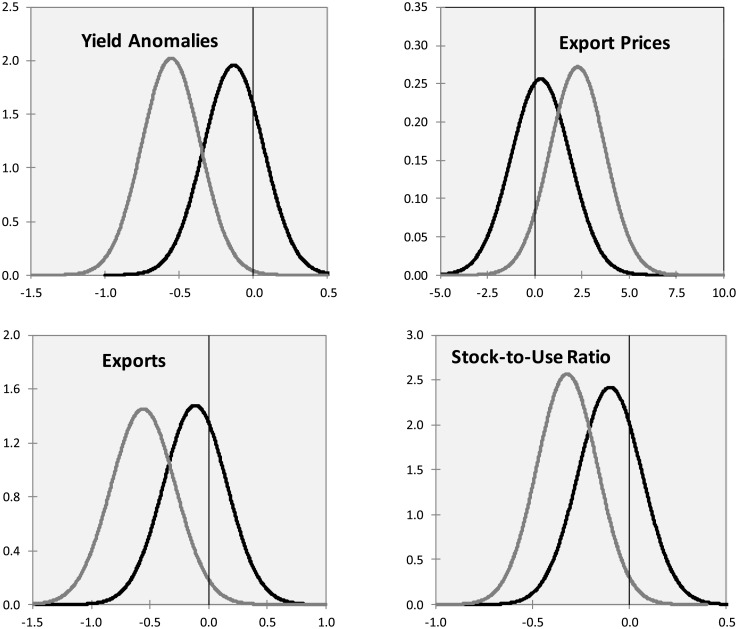
Bootstrapped global impulse response distributions: El Niño (black), La Niña (grey) shocks.

Using Eq (2), we simulate ENSO impulse responses on some endogenous variables introducing in the system one standard deviation shock to the *ElNiño*_*t*_ and *LaNiña*_*t*_ variables. We derive the empirical distribution of impulse responses by employing a sieve bootstrap method with 1000 replications. The endogenous variables are wheat yield anomalies, export price, exports in volume and stock-to-use ratio. Fig 4 summarizes the main results that emerged from the empirical experiment, plotting the distribution of the impact response of the endogenous variables at time 0. La Niña exerts stronger and negative impacts on global yields, exports and stock-to-use ratios than El Niño. Export prices generally increase following an ENSO shock, with La Niña exerting, on average, the highest impact. Normal distributions have been estimated from the 1000 impulse response replications since the Anderson-Darling and Jarque-Bera tests do not reject the null hypothesis that the samples are drawn from this distribution.

Since the GLOWMM allows us to analyze how the shocks influence the endogenous variables and distribute among countries, in the following tables we report the contemporaneous impacts of El Niño and La Niña one standard deviation shocks on various endogenous variables, as their cumulated effects after 1, 3, 6, 12, 24 and 48 months with their statistical significance at one or two standard deviations. We start reporting the estimated median (computed from the 1000 bootstrap replications) impulse responses on wheat yield anomalies. The results, presented in Table 4, show that El Niño events generally have a negative impact on yield. Only Argentina and the EU, among the export countries, show an increase in yields, while Australia experiences the highest negative impact. These phenomena are largely known. [7] report that Argentina’s wheat yield anomalies are positively related to El Niño, with the occurrence of precipitations higher than normal during the season of November-February, while Australia is negatively affected, especially in the east coast, with hot and dry summers and increased frequency of bush fires that cause yield reductions. Focusing on the effects of La Niña on wheat yields, our results generally report a reduction of yields, with the exception of the USA, which shows an increase in the first semester.

**Table 4:** Impact of an El Niño and La Niña shocks on wheat yield anomalies (in percent).

Country	Impact	El Niño					
		Cumulated Responses After					
		1 Month	3 Months	6 Months	12 Months	24 Months	48 Months
USA	-0.16	-0.46*	-0.77*	-0.60	-0.18	0.38	0.77
ARG	0.66	1.35*	1.64*	0.85	0.48	0.81	1.24
AUS	-0.16	-0.96	-3.60*	-4.79*	-3.03	-2.10	-1.27
CAN	-0.83	-1.31*	-2.57*	-2.97*	-1.30	-1.22	-1.06
RUK	-0.06	-0.58	-0.34	1.33	1.70	0.86	0.42
EU	0.07	0.20	0.17	0.40	0.99	1.04	0.92
ROW	-0.07*	-0.03	0.11	0.18	0.16	0.12	0.11
Weighted Average	-0.14	-0.42	-0.85	-0.57	0.11	0.12	0.18
Country	Impact	La Niña					
		Cumulated Responses After					
		1 Month	3 Months	6 Months	12 Months	24 Months	48 Months
USA	0.16	0.41*	0.49	0.26	-0.37	-0.72	-0.91
ARG	-0.48	-0.89	-0.94	-0.49	-0.68	-1.37	-1.79
AUS	-1.53*	-2.11*	-2.67	-1.90	-2.36	-2.40	-1.02
CAN	-1.12**	-2.19**	-2.25*	-1.91*	-2.87*	-3.09*	-2.52
RUK	-0.60*	-1.40*	-1.91*	-2.01	-1.65	-0.97	-0.50
EU	-0.42*	-0.71*	-1.01*	-1.03	-1.21	-1.18	-0.86
ROW	-0.03	-0.08	-0.13	-0.18	-0.11	-0.07	-0.03
Weighted Average	-0.56*	-1.01*	-1.26	-1.18	-1.41	-1.34	-0.96

Notes: Figures are median impulse responses to a one standard deviation increase of *ElNiño*_*t*_ or reduction of *LaNiña*_*t*_ variables. The impact is in percentage points and the horizon is monthly. Symbols ** and * denote significance at 5–95% and 16–84% bootstrapped error bounds respectively.

Given the negative effects on wheat yields, we expect that export prices will be affected by the two ENSO phases. In Table 5, we report the results. They highlight the strong, positive and significant influence exerted by La Niña occurrences. A one standard deviation La Niña weather shock has an impact on global wheat export prices of +2.3%, with a cumulated effect of +7.4% after one year. The exporting countries that appear more reactive are the USA and Australia, which experience an increase in wheat prices of +7.3% and +9.5%, respectively, after one year. El Niño anomalies have a positive impact on wheat export prices. However, the impulse responses are generally not significant at 5% or 16% level. Moreover, starting from the first month, the shapes of El Niño cumulated responses are negative for most export countries.

**Table 5:** Impact of an El Niño and La Niña shocks on wheat export prices (in percent).

Country	Impact	El Niño					
		Cumulated Responses After					
		1 Month	3 Months	6 Months	12 Months	24 Months	48 Months
USA	1.61	0.62	-0.24	-1.45	-2.16	-2.20	-1.58
ARG	2.51*	2.02	0.63	-0.07	-1.25	-1.07	-0.93
AUS	1.13	0.97	1.07	0.03	-1.67	-1.83	-1.46
CAN	-1.57	-0.45	-1.03	-1.74	-2.15	-2.21	-2.20
RUK	0.78	-0.28	-1.48	-3.14	-4.53	-3.62	-2.91
EU	-0.90	-1.82	-2.61	-4.34	-4.75	-3.94	-2.91
Weighted Average	0.32	-0.23	-1.02	-2.36	-3.30	-2.89	-2.30
Country	Impact	La Niña					
		Cumulated Responses After					
		1 Month	3 Months	6 Months	12 Months	24 Months	48 Months
USA	3.59**	5.88**	6.11**	7.54**	7.79**	7.75*	6.47
ARG	1.10	2.56*	3.20*	4.09*	4.30	4.02	2.64
AUS	3.55**	6.69**	7.95**	9.56**	10.14*	9.66*	7.36
CAN	1.82*	3.65*	4.61*	6.28*	6.85*	6.87*	5.81
RUK	1.13	5.90**	7.65*	8.37*	8.68*	8.12*	6.26
EU	2.70*	4.36	5.89*	6.98*	7.29*	6.76*	5.29
Weighted Average	2.31*	5.17**	6.38**	7.57**	7.94*	7.58	6.00

Notes: Figures are median impulse responses to a one standard deviation increase of *ElNiño*_*t*_ or reduction of *LaNiña*_*t*_ variables. The impact is in percentage points and the horizon is monthly. Symbols ** and * denote significance at 5–95% and 16–84% bootstrapped error bounds respectively.

Focusing on wheat exports, in Table 6 we note that global exports are negatively related to ENSO anomalies. As for wheat yields, El Niño exerts a positive, and statistically significant, impact on Argentina’s exports. Canada is negatively affected by La Niña, with a cumulated effect of -5.0% two years after the shock. The group of Russia, Ukraine and Kazakhstan is also negatively affected, and a La Niña shock has a negative impact on worldwide wheat exports, with a cumulated effect of -2.2% during the first year following the shock. Bucking this trend, the US shows strong improvement of exports.

**Table 6:** Impact of an El Niño and La Niña shocks on wheat exports (in percent).

Country	Impact	El Niño					
		Cumulated Responses After					
		1 Month	3 Months	6 Months	12 Months	24 Months	48 Months
USA	-0.27	-0.64	-1.00	-1.52	-2.20	-2.08	-2.08
ARG	3.24**	6.04**	7.59*	6.94*	6.97*	5.75	5.49
AUS	0.30	0.48	-0.79	-1.71	-0.35	0.63	1.09
CAN	-0.35	-1.35*	-3.00*	-3.88*	-2.91	-2.50	-2.25
RUK	-0.13	-1.34	-0.38	2.54	2.48	0.30	-0.84
EUR	-0.10	-0.34	0.30	1.85	4.00	3.93	2.75
ROW	-1.34*	-2.02*	-2.13	-1.87	-3.99	-3.68	-4.17
Weighted Average	-0.13	-0.62	-0.62	0.12	0.47	0.08	-0.42
Country	Impact	La Niña					
		Cumulated Responses After					
		1 Month	3 Months	6 Months	12 Months	24 Months	48 Months
USA	1.10**	3.37**	5.56**	6.58**	7.04**	7.18*	6.67*
ARG	-0.95	-0.38	0.99	2.81	4.44	6.03	5.51
AUS	-0.87*	-1.08*	-2.57	-2.51	-2.92	-2.36	-1.10
CAN	-1.29**	-2.30**	-2.83**	-3.14*	-4.49*	-4.97*	-4.83*
RUK	-2.50	-7.03**	-10.73**	-12.46*	-11.22*	-10.01*	-9.24
EU	0.13	-0.26	-0.99	-0.86	-1.06	-1.19	0.16
ROW	1.77**	3.45**	5.41**	6.24*	7.69*	8.01*	7.38
Weighted Average	-0.56	-1.39	-2.10	-2.23	-1.89	-1.49	-1.04

Notes: Figures are median impulse responses to a one standard deviation increase of *ElNiño*_*t*_ or reduction of *LaNiña*_*t*_ variables. The impact is in percentage points and the horizon is monthly. Symbols ** and * denote significance at 5–95% and 16–84% bootstrapped error bounds respectively.

Finally, in Table 7 we show that a one standard deviation shock to El Niño after one year produces an increase of 2% of the stock-to-use ratio rate in the US. The impulse responses are also positive for Argentina and the EU. The strongest negative effect is observed for Canada, where the stock-to-use ratio is always significantly negative, with a reduction of 1.7 percentage points. On average, the effect of El Niño is negative (but not significant). In accordance with the previous results, many countries exhibit a significant reduction of the stock-to-use ratio as result of La Niña shocks.

**Table 7:** Impact of an El Niño and La Niña shocks on stock-to-use ratio.

Country	Impact	El Niño					
		Cumulated Responses After					
		1 Month	3 Months	6 Months	12 Months	24 Months	48 Months
USA	0.10	0.16*	0.45**	0.90*	1.87*	2.48*	2.80
ARG	0.06*	0.15*	0.32	0.43	0.23	-0.47	-0.51
AUS	-0.27*	-0.89*	-1.98	-2.94	-2.60	-2.71	-2.73
CAN	-0.54*	-1.33*	-3.03*	-2.87	-1.06	-1.13	-1.18
RUK	-0.10	-0.15	-0.43	-0.94	-1.09	-0.84	-0.70
EU	0.09*	0.15	0.20	0.07	0.09	0.11	0.09
ROW	-0.07	0.16	0.45	0.73	0.95	0.86	0.70
Weighted Average	-0.10	-0.24	-0.57	-0.71	-0.30	-0.20	-0.14
Country	Impact	La Niña					
		Cumulated Responses After					
		1 Month	3 Months	6 Months	12 Months	24 Months	48 Months
USA	-0.02	-0.31	-1.09*	-1.83*	-2.70*	-3.18*	-2.78
ARG	-0.03	-0.08	-0.08	0.12	0.83	1.53	1.31
AUS	-1.13*	-1.95*	-1.86	-2.35	-3.02	-3.68	-3.68
CAN	-1.72**	-2.94**	-3.43**	-3.59*	-4.44*	-4.38*	-3.84*
RUK	0.27**	0.60**	0.85*	1.07*	1.20	1.07	0.82
EU	-0.13*	-0.33**	-0.50*	-0.58**	-0.56	-0.58	-0.50
ROW	0.01	-0.36*	-0.80*	-1.07*	-1.13*	-1.22	-1.26
Weighted Average	-0.32*	-0.62*	-0.81*	-0.99	-1.25	-1.41	-1.34

Notes: Figures are median impulse responses to a one standard deviation increase *ElNiño*_*t*_ or reduction *LaNiña*_*t*_ variables. The impact is in percentage points and the horizon is monthly. Symbols ** and * denote significance at 5–95% and 16–84% bootstrapped error bounds respectively.

## Robustness checks

Export shares drastically changed during the years 2000-2016. Table 8 reports the values. The highest export share is now shown by Black Sea countries, accounting for 25.5% of the world’s wheat exports during 2014-2016 (approximately 45 millions of metric tons in 2016 marketing year). In 2000, the share was only 11%. The USA has seen its share reduce from 26.6% in the 2000 marketing year to 15.2% in the 2016 marketing year. Minor reductions are shown by Canada and Australia, while the EU increased its export share during the period 2000-2016. The choice of trade weights to aggregate single country/region models is based on the rationale that the exogenous shocks, including ENSO shocks, could be passed on to all countries through trade channels. Thus, we check whether possible changes of trade shares to aggregate country’s models and to generate the foreign variables may affect the impulse responses.

**Table 8:** Wheat export shares (percentage).

Country	Periods			
	2000.7–2005.6	2005.7–2010.6	2010.7–2014.6	2014.7–2016.6
ARG	9,72	6,51	4,95	3,97
AUS	14,65	11,97	13,11	11,26
CAN	14,60	14,95	13,47	13,45
RUK	10,94	20,49	19,49	25,51
EU	12,86	13,54	14,45	19,57
USA	26,61	23,29	21,80	15,23
ROW	10,61	9,25	12,71	11,02
WORLD	100,00	100,00	100,00	100,00

Notes: USDA, Grain: World Markets and Trade Dataset

**Fig 5:**
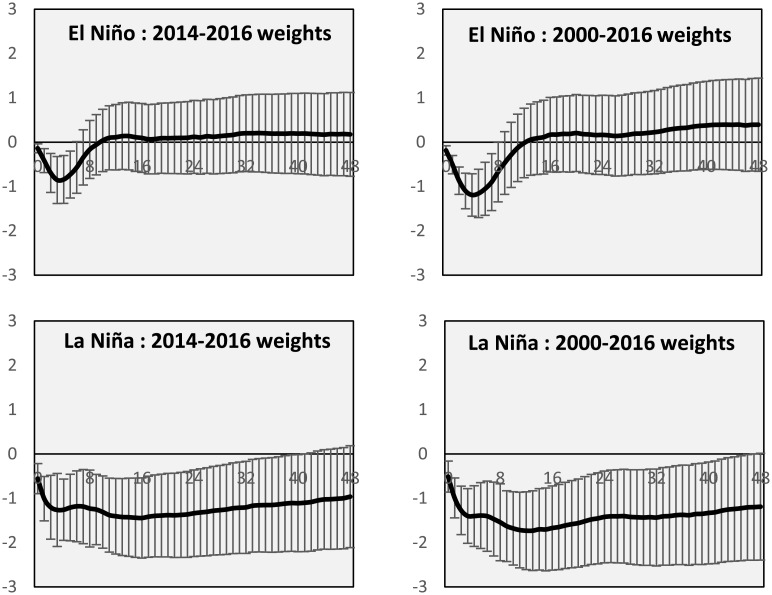
Varying wheat trade weights, El Niño and La Niña: Yield anomalies and error bars impulse responses.

**Fig 6:**
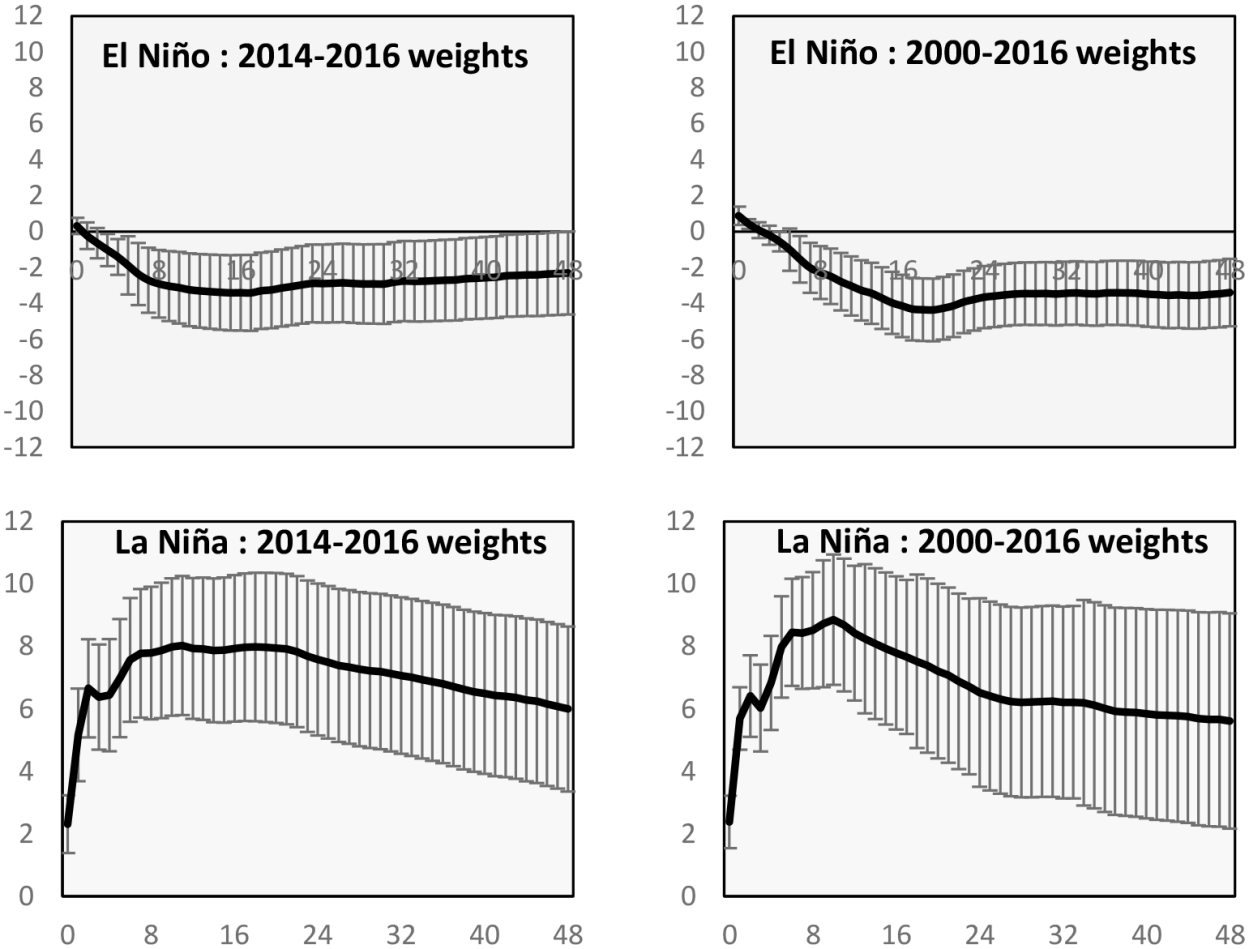
Varying wheat trade weights, El Niño and La Niña: Export prices and error bars impulse responses.

In Figs 5 and 6, we present the impulse responses for the endogenous variables of wheat yield and export prices calculated through the use of the average weights during the period 2000–2016 and for the period 2014–2016. For both cases, i.e. El Niño and La Niña events, we report the median impact on the global wheat market and one standard error bars. From the Figs 5 and 6 it emerges that while the shapes are similar, some differences are noted. The cumulated effects of impulse responses for the model estimated with 2000–2016 weights are generally higher than those computed with the trade weights for 2014–2016. However, this is not true for export prices, where the long-run response to a La Niña shock is higher using the latter weights. This simulation study underlines the importance of updating the trade weights in order to take into account possible trade shifts and the way shocks transmit across the world wheat market.

**Fig 7:**
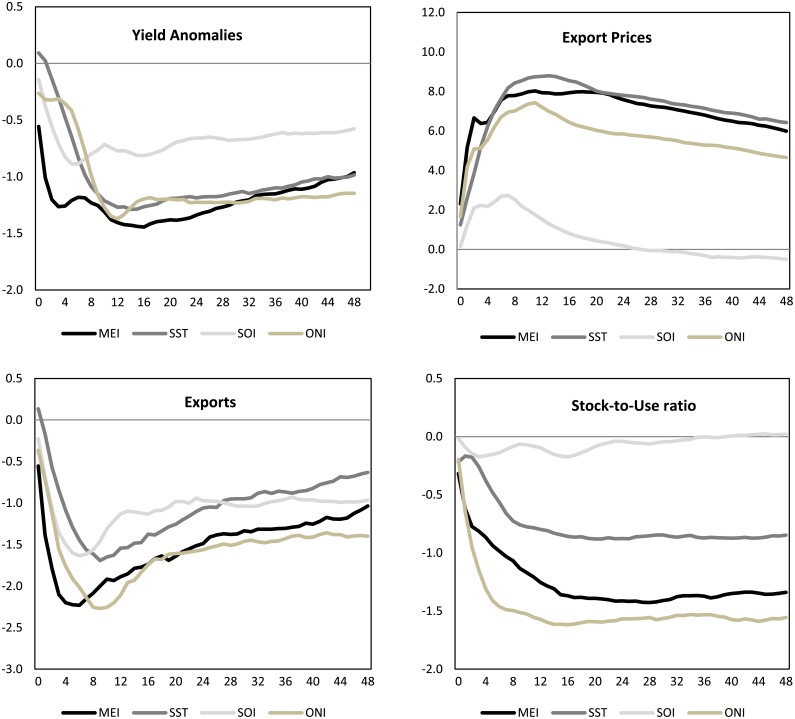
La Niña IR responses: MEI, SST, ONI and SOI indicators.

In the second experiment, we check the robustness of the results using different ENSO indicators, specifically the SST, the SOI and the ONI indicators. Since the MEI indicator is constructed as a bi-monthly average, we replace the raw variables SST and SOI with their bi-monthly recursive averages (the ONI indicator is given by the three-monthly average of the SST indicator). We standardize all the variables using the 2000–2016 average and standard deviation values. Since La Niña phase seems to have more influence than an El Niño event, in Fig 7 we present the impact and cumulated impulse responses to one standard deviation shock of each of the four indicators—MEI, SST and SOI, plus the ONI indicator—during a La Niña phase.

The impulse responses generally showed similar signs and shapes. However, differences of magnitude could be noted. Among the four indicators, the lowest impact was reported by the SOI indicator, especially in the case of export prices and stock-to-use endogenous variables. The MEI, SST and ONI indicators showed smaller differences. This result introduces the importance of choosing the ENSO indicator. In the following Table 9, we present the contemporaneous correlation matrix of the four indicators.

**Table 9:** Contemporaneous correlation matrix of the MEI, SST, ONI and SOI indicators during El Niño, La Niña phases: 2000.7-2016.6.

	El Niño phase			
	MEI	SST	ONI	SOI
MEI	1	-	-	-
SST	0.89	1	-	-
ONI	0.86	0.94	1	-
SOI	0.62	0.61	0.61	1
	La Niña phase			
	MEI	SST	ONI	SOI
MEI	1	-	-	-
SST	0.92	1	-	-
ONI	0.84	0.87	1	-
SOI	0.79	0.74	0.69	1

As expected by the visual inspection in Fig 1, the lowest correlation coefficients were those included in the last row, i.e. for the correlations between the SOI indicator and the set of the other ENSO indicators. The lowest values were reported during the El Niño phase and with respect to the SST and ONI indicators. Since the SOI is based on the sea level pressure at just two individual stations (Tahiti and Darwin, Australia), it can be affected by short-term fluctuations unrelated to ENSO. However, averaging the SOI indicator over months may help to consider for relevant deviations, like those associated with ENSO. Another limitation of the SOI index is that, while MEI, SST and ONI indexes are measured in relatively the same region (i.e. the equatorial Pacific closer to central and eastern Pacific), the SOI measurements are related to the south of the equatorial Pacific. This may alter the ENSO signal [14] and thus explain the differences in the set of estimated parameters, the diverse shapes of the impulse responses and their higher variability. In this respect, since we focused our analysis on the MEI composite indicator, the analysis and results can be protected from possible biased signals produced by one single ENSO indicator. However, further studies are needed to explore this important field of analysis.

## Conclusion

In this study, we analyze the El Niño-Southern Oscillation (ENSO) climate event to investigate the effect of its shocks on the worldwide wheat market. We estimated a Global VAR (GVAR) model for the six main export regions, plus the rest of the world region. Unlike other studies that analyze the transmission of ENSO shocks using home-country models, which do not allow for international interactions, we employ a dynamic multi-region model. This strategy allows us to take into account economic interlinkages and spillovers across the world wheat market. Further, the model permits the interplay of other important determinants such as exchange rates, stock-to-use ratios, fertilizer prices, food prices and oil prices, thereby disentangling ENSO anomalies from other possible sources.

In synthesis, our results show that in both the short run and long run, El Niño shocks have smaller effects on important endogenous variables in the system, such as wheat yields, export prices, exports and stock-to-use ratio than La Niña occurrences. At a global level, after El Niño or La Niña shocks, we report a decrease of yields, exports and stock-to-use ratios, while wheat export prices rise. Using the model, we are able to decompose the global shocks into impacts and cumulated effects on individual countries/regions. Some interesting behaviours can be noted. Following an El Niño shock, Argentina and the EU show an increase in wheat yields, while Australia, as expected, shows a reduction of wheat yields. The effects of La Niña on yield anomalies are negative for all countries, with the exception of the US, which shows an increased yield in the first six months.

La Niña exerts important effects on wheat prices, especially in the US and Australia. One year after the initial shock, the impulse responses of the two countries are +7.8% for the US and +10.1% for Australia. By contrast, in the long run, El Niño seems to have a negative (but not statistically significant) cumulated effect on wheat export prices. Focusing on exports, Argentina is the country that experiences an increase in exports following El Niño events, as with the US in the case of La Niña events. A one standard deviation El Niño shock produces an increase of 2% of the stock-to-use ratio rate after one year in the US. The impulse responses are also positive for Argentina and the EU, while the strongest negative effect is observed for Canada, where the stock-to-use ratio is always significantly negative.

The coverage of research can be enlarged in different ways. One of the more promising prospects is to include a climate model in the system. The El Niño-Southern Oscillation has a large influence on seasonal precipitation and temperature patterns across the globe. A forecast procedure of ENSO effects on precipitation and temperature can help in designing the future dynamics of yields and wheat export prices, exports and stocks for the main export countries. A complete model will improve the ability of national governments to introduce policies that will allow them to reduce and manage wheat price volatility, minimizing the possible loss of consumer and producer surplus. We believe that further studies are needed to explore this important field of analysis. We leave these tasks for future research.

## Supporting information

S1 Appendix (PDF)

## References

[1] Ropelewski C, Halpert M. Global and Regional Scale Precipitation Patterns Associated with the El Niño/Southern Oscillation. Monthly Weather Review. 1987;115: 1606–1626. 10.1175/1520-0493(1987)115<1606:GARSPP>2.0.CO;2

[2] Ropelewski C, Halpert M. Precipitation patterns associated with the high index phase of the Southern Oscillation. Journal of Climate. 1989;2: 268–284. 10.1175/1520-0442(1989)002<0268:PPAWTH>2.0.CO;2

[3] Brönnimann S. Impact of El Niño-Southern Oscillation on European climate. Reviews of Geophysics. 2007;45: 1–28.

[4] Shaman J, Tzieperman E. An Atmospheric Teleconnection Linking ENSO and Southwestern European Precipitation. Journal of Climate. 2011;24: 124–139. 10.1175/2010JCLI3590.1

[5] Hammer GL, Nicholls N, Mitchell C. Applications of Seasonal Climate Forecasting in Agricultural and Natural Ecosystems. New York: Springer; 2000.

[6] Rosenzweig C, Hillel D. Climate Variability and the Global Harvest. Oxford: Oxford University Press; 2008.

[7] Iizumi T, Luo JJ, Challinor AJ, Sakurai G, Yokozawa M, Sakuma H, et al Impacts of El Niño Southern Oscillation on the Global Yields of Major Crops. Nature Communications 2014;5: 904–908. 10.1038/ncomms4712 24827075

[8] Brunner AD. El Niño and world primary commodity prices: Warm water or hot air? Review of Economics and Statistics. 2002;84: 176–183.

[9] Laosuthi T, Selover DD. Does El Niño Affect Business Cycles? Eastern Economic Journal. 2007;33: 21–42.

[10] Cashin P, Mohaddes K, Raissi M. Fair weather or foul? The macroeconomic effects of El Niño. Journal of International Economics. 2017;106: 37–54. 10.1016/j.jinteco.2017.01.010

[11] Ubilawa D. El Niño, La Niña and world coffee price dynamics. Agricultural Economics. 2012;43: 17–26. 10.1111/j.1574-0862.2011.00562.x

[12] Ubilawa D, Holt M. El Niño southern oscillation and its effects on world vegetable oil prices: assessing asymmetries using smooth transition models. The Australian Journal of Agricultural and Resource Economics. 2013;57: 273–297. 10.1111/j.1467-8489.2012.00616.x

[13] Gutierrez L, Piras F, Roggero PP. A Global Vector Autoregression Model for the Analysis of the Wheat Export Prices. American Journal of Agricultural Economics. 2015; 97: 1494–1511. 10.1093/ajae/aau103

[14] Barnston A. Why are there so many ENSO indexes, instead of just one? NOAA, Climate.gov. 2015;1–5. Available from: https://www.climate.gov/news-features/blogs/enso/why-are-there-so-many-enso-indexes-instead-just-one.

[15] Mazzarella A, Giuliacci A, Scafetta N. Quantifying the Multivariate ENSO Index (MEI) coupling to CO2 concentration and to the length of day variations. Theoretical and Applied Climatology. 2013;111: 601–607 10.1007/s00704-012-0696-9

[16] Nicholls N. Impact of the Southern Oscillation on Australian Crops. Journal of Climatology. 1985;5: 553–560. 10.1002/joc.3370050508

[17] Legler D, Bryant K, O’Brien J. Impact of ENSO-related Climate Anomalies on Crop Yields in the U.S. Climatic Change 1999;42: 351–375. 10.1023/A:1005401101129

[18] Rosenzweig C., Iglesias A., Yang X., Epstein PR, Chivian E. Climate Change and Extreme Weather Events: Implications for Food Production, Plant Diseases, and Pests. Global Change & Human Health. 2001;2: 90–104. 10.1023/A:1015086831467

[19] Selvaraju R. Impact of El Niño–Southern Oscillation on Indian Foodgrain Production. International Journal of Climatology. 2003;23: 187–206. 10.1002/joc.869

[20] Mallya G, Zhao L, Song X, Niyogi D, Govindaraju R. 2012 Midwest Drought in the United States. Journal of Hydrologic Engineering. 2013;18: 737–745. 10.1061/(ASCE)HE.1943-5584.0000786

[21] Wolter K, Timlin MS. Monitoring ENSO in COADS with a seasonally adjusted principal component index. Procedures of the 17th Climate Diagnostics Workshop, Norman, OK, Univ. of Oklahoma. 1993; 52–57.

[22] Keppenne C. AAn ENSO signal in soybean futures prices. Journal of Climate. 1995;8: 1685–1689. 10.1175/1520-0442(1995)008<1685:AESISF>2.0.CO;2

[23] Hodrick R, Prescott EC. Postwar U.S. Business Cycles: An Empirical Investigation. Journal of Money, Credit, and Banking. 1997;29: 1–16. 10.2307/2953682

[24] Pesaran MH, Schuermann T, Weiner S. Modelling regional interdependencies using a global error-correcting macroeconometric model. Journal of Business and Economics Statistics. 2004;22: 129–162. 10.1198/073500104000000019

[25] Dées S, Di Mauro F, Pesaran MH, Smith LV. Exploring the international linkages of the euro area: A global VAR analysis. Journal of Applied Econometrics. 2007;22: 1–38. 10.1002/jae.932

[26] Di Mauro F, Pesaran MH The GVAR handbook: Structure and applications of a macro model of the global economy for policy analysis. Oxford: Oxford Economic Press; 2013.

[27] Lütkepohl H. New introduction to multiple time series analysis. Berlin: Springer; 2005.

[28] Ravn MO, Uhlig H. On adjusting the Hodrick-Prescott filter for the frequency of observations. Review of Economics and Statistics. 2002;84: 371–376. 10.1162/003465302317411604

[29] Gutierrez L. Speculative bubbles in agricultural commodity markets. European Review of Agricultural Economics. 2013; 40(2): 217–238. 10.1093/erae/jbs017

[30] Johansen S. Cointegration in Partial Systems and the Efficiency of Single-Equation Analysis. Journal of Econometrics. 1992;52: 231–54. 10.1016/0304-4076(92)90019-N

[31] Johansen S. Likelihood-Based Inference in Cointegrated Vector Autoregressive Models. Oxford: Oxford University Press; 1995.

[32] Godfrey LG. Testing against general autoregressive and moving average error models when the regressors include lagged dependent variables. Econometrica. 1978;46: 1293–1302. 10.2307/1913830

[33] Godfrey LG. Testing for higher order serial correlation in regression equations when the regressors include lagged dependent variables. Econometrica. 1978;46: 1303–1310. 10.2307/1913830

[34] Pesaran MH, Shin Y, Smith R. Structural analysis of vector error correction models with exogenous *I*(1) variables. Journal of Econometrics. 2000; 97:293–343. 10.1016/S0304-4076(99)00073-1

[35] Ploberger W, Krämer W. The CUSUM test with OLS residuals. Econometrica, 1992;60: 271–286. 10.2307/2951597

[36] Nyblom J. Testing for the constancy of parameters over time. Journal of the American Statistical Association. 1989;84: 223–230. 10.1080/01621459.1989.10478759

