# Supplementary material for: Impacts of El Niño-Southern Oscillation on the wheat market: A global dynamic analysis
Source: PLoS One. 2017 Jun 8;12(6):e0179086. doi: 10.1371/journal.pone.0179086 (PMC5464633; doi:10.1371/journal.pone.0179086)
Supplement: S1 Appendix — (PDF) [file pone.0179086.s001.pdf]

Supporting Information

S1 Appendix. Dataset. In this appendix we describe the data sources and key steps in the analysis of data.

Wheat Export Prices:

Argentina: Trigo Pan wheat up river;

Russia: Black Sea milling wheat;

Australia: ASW wheat Eastern States;

Canada: CWAD wheat St Lawrence;

EU: France standard grade wheat Rouen;

USA: SRW wheat Gulf.

All prices are denoted in US dollar per ton.

Index : $2000.7 - 2001.6 = 100$.

Source: FAO, Food Price and Analysis Tool.

Stock to utilization ratio

Ratio of estimated Ending Stocks (thousand metric tons) on estimated

Consumption (thousand metric tons).

Source: USDA, Grain World Markets and Trade.

Yield

Ratio of estimated wheat Production (thousand metric tons) on estimated

Wheat Harvested Area (thousand hectares).

Source: USDA, Grain World Markets and Trade, various years.

Wheat Exports

Thousand metric tons.

Index : $2000.7 - 2001.6 = 100$.

Source: USDA, Grain World Markets and Trade.

Nominal Exchange rates

Nominal exchange rate : Local currency per unit of US dollar;

Argentina: Pesos;

Russia: Rublo;

Australia: Australian dollar;

Canada: Canadian dollar;

EU: Euro;

Rest of World: Weighted average of Brasil: Reals; China: Yuan;India: Rupees;

Mexico: Pesos; Turkey: Liras;

Weight are given by the wheat production of each country on the total production of these countries.

Index : 2000.7 – 2001.6 = 100.

Source : IMF Financial Statistics and Financial Statistics of the Federal Reserve Board.

Fertilizer price

DAP (Diammonium Phosphate) price.

Nominal US dollar per metric tons.

Price transformed in local currency using the local exchange rate against the US dollar.

Index : 2000.7 – 2001.6 = 100.

Source: World Bank Commodity Price Data (Pink Sheet).

Oil price

Crude oil price.

Nominal US dollar per barrel.

Index : 2000.7 – 2001.6 = 100.

Source: World Bank Commodity Price Data (Pink Sheet).

Food consumption prices

Index : 2000.7 – 2001.6 = 100.

Source: Eurostat and National Statistics

Export weights

Computed from Grain: World Markets and Trade, various years.

Metric tons.

Source: USDA.
